# The Cell Wall Teichuronic Acid Synthetase (TUAS) Is an Enzyme Complex Located in the Cytoplasmic Membrane of *Micrococcus luteus*

**DOI:** 10.1155/2010/395758

**Published:** 2010-04-06

**Authors:** Lingyi Lynn Deng, Alice A. Alexander, Sijin Lei, John S. Anderson

**Affiliations:** ^1^Department of Biochemistry, Molecular Biology and Biophysics, University of Minnesota, St. Paul, MN 55108, USA; ^2^Research Service, VA Boston Healthcare System, Boston, MA 02130, USA; ^3^Department of Medicine, Boston University School of Medicine, 650 Albany Street, X-Rm808, Boston, MA 02118, USA; ^4^St. Jude Medical, Inc., St. Paul, MN 55117, USA

## Abstract

The cell wall teichuronic acid (TUA) of *Micrococcus luteus* is a long-chain polysaccharide composed of disaccharide repeating units [-4-*β*-D-ManNAcA*p*-(1→6)*α*-D-Gl*cp*−1-]_*n*_, which is covalently anchored to the peptidoglycan on the inner cell wall and extended to the outer surface of the cell envelope. An enzyme complex responsible for the TUA chain biosynthesis was purified and characterized. The 440 kDa enzyme complex, named teichuronic acid synthetase (TUAS), is an octomer composed of two kinds of glycosyltransferases, Glucosyltransferase, and ManNAcA-transferase, which is capable of catalyzing the transfer of disaccharide glycosyl residues containing both glucose and the *N*-acetylmannosaminuronic acid residues. TUAS displays hydrophobic properties and is found primarily associated with the cytoplasmic membrane. The purified TUAS contains carotinoids and lipids. TUAS activity is diminished by phospholipase digestion. We propose that TUAS serves as a multitasking polysaccharide assembling station on the bacterial membrane.

## 1. Introduction

**Figure 1:**
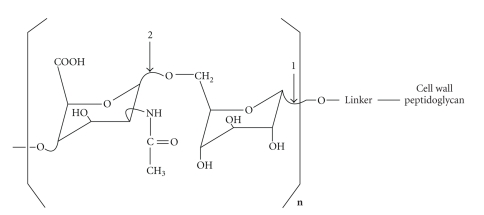
Chemical structure of teichuronic acid in the cell wall of *Micrococcus luteus *and proposed site of action for glucosyltransferase (indicated by arrow 1) and that for ManNAcA transferase (indicated by arrow 2). The linker contains an oligosaccharide with a GlcNAc residue at the reducing end and a phosphate.

*Micrococcus luteus *is a typical gram-positive bacterium. The cell wall teichuronic acid (TUA) is a long chain polysaccharide composed of almost a hundred disaccharide repeating units. TUA plays an important role in protecting the microbe and interacting with the host cells. TUA consists of D-glucose and *N-*acetyl-D-mannosaminuronic acid (ManNAcA) residues, in which the structural repeat unit is [→4)ß-D-ManNAcA*p*-(1→6)*α*-D-Glc*p* -(1→]n [1, 2] (Figure 1). It is located on the cell surface and is covalently linked to peptidoglycan in *M. luteus* cell walls through a linker. Although experimental transfer of linkage oligosaccharide (or growing TUA chain) from carrier lipid to peptidoglycan remains to be demonstrated, the linkage region is found to be composed of a phosphate and an oligosaccharide as shown in Figures 1 and 6 [3, 4]. In vitro initial (de novo) synthesis of teichuronic acid by cytoplasmic membrane fragments requires uridine diphosphate *N-*acetyl-D-glucosamine (UDP-GlcNAc), uridine diphosphate *N-*acetyl-D-mannosaminuronic acid (UDP-ManNAcA), and UDP-glucose [3]. During biosynthesis, UDP-GlcNAc is the donor of the initial residue, which becomes the potentially reducing terminal residue of native teichuronic acid. However, wall-membrane preparations require only UDP-glucose and UDP-ManNAcA to effect elongation of teichuronic acid already present in the wall fragments [5]. Similarly, if exogenous soluble teichuronic acid is supplied as acceptor, UDP-glucose and UDP-ManNAcA are codependently required by detergent solubilized extracts of cytoplasmic membrane fragments to effect significant elongation of the acceptor teichuronic acid [6]. Transfer of glucosyl residues from UDP-glucose to teichuronic acid is catalyzed by glucosyltransferase and the addition of *N-*acetylmannosaminuronic acid residues is catalyzed by the ManNAcA-transferase (Figure 1). Very little is known about how these two enzymes are arranged and located, what other protein factors are also involved in the process of the polysaccharide biosynthesis, and how this extracellular polysaccharide is synthesized, elongated, and transported.

Membrane preparations of *Micrococcus luteus* are easily prepared by lysozyme digestion and solubilization of the cell wall. Protoplasts are generated if the lysozyme treatment is done in osmotically stabilizing medium and membrane fragments or ghosts are produced in hypotonic medium. Because of the ease of cell wall removal, *M. luteus* constitutes an excellent model system for studying the structural organization of membrane components as well as investigation of membrane function [7]. The most extensively characterized enzyme of the membrane of this organism is the F_1_-ATPase which constitutes up to 10% of total membrane protein. 

Little is known about the membrane placement of polymer synthesizing enzymes or how they participate in the vectorial process, in which a substrate produced in the cytoplasm is utilized for synthesis of an external polymer. To initiate an investigation of such vectorial relationships, we have examined the cytological localization of an enzyme involved in the biosynthesis of teichuronic acid in *M. luteus,* namely, the teichuronic acid synthetase.

In our early study of cell wall biosynthesis, cytoplasmic membrane fragments, solubilized with the detergents Thesit and CHAPS, were a source of glycosyltransferase. The enzyme was purified about 200-fold, and found to be a 440 kDa protein, which was responsible for TUA elongation. The purified native enzyme was a multisubunit protein consisting of subunits of two sizes (four copies of each); their molecular masses were determined to be ~52.5 and ~54 kDa. Since the radioactive assay is based on measuring the incorporation of [^14^C]glucose from UDP-[^14^C]glucose into TUA polymer, we tentatively classified this protein as a Glucosyltransferase [8]. In the current study, the same protein was further characterized by analyzing the physical and chemical properties as well as the subcellular locations. The additional biological function of this enzyme complex was discovered and a topological model is proposed.

## 2. Materials and Methods

### 2.1. Growth of Bacteria, Preparation, and Isolation of Cellular Fractions

Cultures of *Micrococcus luteus *(ATCC 4698) were grown at 30°C in a vigorously shaken liquid culture medium containing peptone (10 g/liter) and NaCl (5 g/liter). The cells were usually harvested in the mid-log phase. 

In general, the crude cell extract (bacterial lysate) was obtained by digesting the cell pellet with lysozyme, and then with DNase and RNase. The membrane fraction was obtained by centrifugation between 3,000 and 12,000 ×g. The cytoplasmic membrane fragments were recovered from the supernatant by further centrifugation at 48,000 ×g for l hour. The final pellet was washed several times and solubilized in a buffer containing detergents, Thesit, and CHAPS, with glycerol and magnesium ion to stabilize the solubilized enzyme [8].

To determine the TUAS activity in the different subcellular compartments, a specific experiment was conducted in which crude cell extract was prepared by physically grinding cells with alumina instead of lysozyme digestion [5]. Thus four fractions were obtained including (1) cytosol, (2) cytoplasmic membrane, (3) the cell wall, and (4) the culture supernatant or extracellular cell filtrate. The protein concentration in each fraction was standardized to 1 *μ*g/ml before the TUAS assay.

The *cytosol fraction* was prepared by filtering crude cell extract through two 0.22 mm Millipore nitrocellulose filters under vacuum.

The *wall-membrane* fraction was recovered by a slower speed centrifugation (3,000 to 12,000 ×g) of a crude extract. The wall-membrane fraction was loaded onto a sucrose step gradient consisting of equal volumes of 14, 16, and 17% sucrose and centrifuged at 18,000 rpm. The *cell wall fraction* was collected from the bottom of the gradient and the *membrane fraction* from the region of the junction between 14 and 16% sucrose layers and was further solubilized by the detengent Thesit (50 mg/ml) and CHAPS (0.5 mg/ml) [6]. 

The *culture supernatant* was precipitated by addition of ammonium sulfate to a concentration of ~0.25 g/ml and collected by centrifugation at 12,000 ×g for 25 minutes. The precipitate was resuspended and dialyzed against the same buffer. Extraneous ions were removed by passage of the sample through an ion retardation column (Bio-Rad).

### 2.2. Purification of the Enzyme Complex

The following steps were used to purify TUAS from detergent-solubilized extract or the membrane fraction: adsorbent column chromatography (Bio-Bead SM-2, flow through), anionic ion exchange chromatography (DEAE-cellulose column, eluted with 0.25 M NaCl), size exclusion chromatography (Bio-Gel P-300 column, exclusion/void volume), isoelectric precipitation (pH 5.5), ammonium sulfate fractionation (top layer), adsorbent column, and preparative gradient polyacrylamide gel electrophoresis [8]. 

### 2.3. Enzymatic Assays of TUAS

TUAS activity was determined by incubating 50 *μ*l of enzyme sample (liquid sample or gel slices) and 50 *μ*l of reaction mix at 25° for 60 minutes. The complete reaction mixture contained 0.5–0.7 mM UDP-[^14^C]glucose (2 *μ*Ci/umol), 0.5–0.7 mM UDP-ManNAcA, 50 mM HEPES pH 8.2, 20 mM magnesium acetate, 2 mM 2-mercaptoethanol, 9 to 35 mg of cell wall acceptor per ml, and 0.15 to 15 *μ*g protein per ml. The cell wall acceptor refers to the purified TUA preparation (a short, soluble form of TUA) which was originally released from the *M. luteus* cell wall by treatment with acid. This soluble acceptor was found to be capable of accepting glucose and ManNAcA and facilitating the elongation of the TUA chain [6]. 

The reaction was stopped by adding 20–30% (v/v) concentrated isobutyric acid. The radioactive product was separated by paper chromatography in isobutyric acid: 1 M NH_4_OH (5 : 3 v/v). The product at the origin of the paper was quantitated by liquid scintillation counting. It had previously been analyzed by mass spectrometry, nuclear magnetic resonance imaging (NMR), and carbohydrate PAGE and had been confirmed to be teichuronic acid [2, 9]. In addition, the enzymatic products were also analyzed with a preparative nonreducing polyacrylamide gel electrophoresis followed either by alcian blue/silver staining or autoradiography [6, 8, 10]. 

### 2.4. Physical and Chemical Properties of TUAS

The effect of different incubating conditions and reagents on the TUAS enzyme activity were studied (such as enzyme substrates, growth phases, detergents, and phospholipase treatment). Other physical and chemical properties of purified TUAS were also studied including density, hydrophobicity, mobility, optical properties, isoelectric point, physical form, and subcellular distribution.

### 2.5. Lipids and Pigment Analysis

The optical properties (including pigments) of the purified active fraction(s) were analyzed by spectrophotometry, with wavelengths ranging from 200 nm to 700 nm. The same fractions were also subjected to phase partition with solvent mix (Chloroform : Methanol : distilled water; 8 : 4 : 3, v/v/v). The lower organic phase was extracted and per-methylated. The chemically derivatized product was analyzed on a GC-MS (HP 6890 gas chromatograph/HP5973 MSD) with a DB-17 capillary column at a constant flow rate of 1 ml/min [11].

### 2.6. Immunologic Assays

Antiserum to TUAS was raised in New Zealand White rabbits. Immunoblot analysis (Western blot) was performed. Immunofluorescence labeling was performed by incubating cells of *M. luteus *with primary antibody (serum) followed by goat antirabbit IgG-FITC conjugate. Labeled cells were observed with a differential phase contrast fluorescent microscope. 

## 3. Results

### 3.1. TUAS Is a Bifunctional Glycosyltransferase

**Figure 2:**
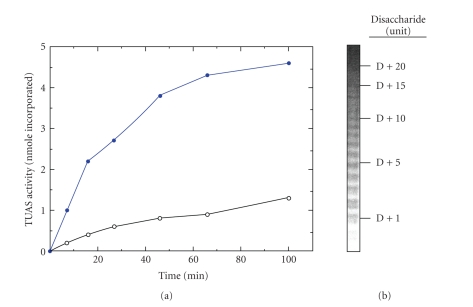
(a) Effect of the two substrates (UDP-[^14^C]glucose and UDP-ManNAcA) on the activity of teichuronic acid synthetase (TUAS). Open circle: with one substrate 0.5 mM UDP-C^14^-Glucose; Closed circle: two substrates by adding 0.5 mM UDP-ManNAcA to the same mix. (b) Analysis of teichuronic acid, the enzymatic product of TUAS, by nondenaturing PAGE stained with Alcian blue and silver. The numbers on the right represent the repeats of the basic TUA disaccharide unit. D is an oligosaccharide of TUA estimated to have 6–10 disaccharide repeats, which is not detectable by PAGE.

The purified enzyme fraction displayed the typical reaction for glucosyltransferase, which is the transfer of [^14^C]glucose from UDP-[^14^C]glucose to the teichuronic acid acceptor. However, the enzyme is also capable of incorporating ManNAcA into the same polysaccharide. Adding 0.5 mM UDP-ManNAcA in addition to 0.5 mM UDP-glucose can significantly increase the rate of teichuronic acid biosynthesis by ~400% (Figure 2(a)). The enzymatic products were analyzed by Native-PAGE, which revealed a typical ladder pattern for linear polysaccharide with one repeating unit or two sugars apart (Figure 2(b)). 

The GC-MS analysis of the same enzymatic product hydrolyzed with trifluoroacetic acid (TFA) showed two component sugars: Glucose and ManNAcA. These results indicated that efficient biosynthesis and elongation of teichuronic acid polymer by purified TUAS requires both UDP-ManNAcA and UDP-Glucose. Or in other words, TUAS is a bifunctional glycosyltransferase capable of transferring both the component sugars (Glucose and ManNAcA) for the TUA polymer.

### 3.2. TUAS Is a Multi-Subunit Enzyme Complex

**Figure 3:**
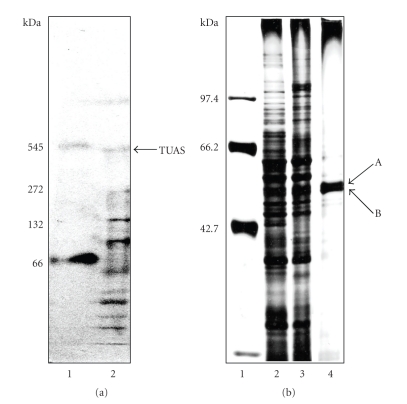
(a) *M. luteus* TUAS analyzed with native gradient PAGE. Partially purified TUAS was subjected to electrophoresis on 3–12% native gradient polyacrylamide gels (left, lane 2). Protein bands were visualized by staining with Coomassie blue. The molecular-mass standards of 545, 272, 132, and 66 kDa (top to bottom) are shown (left, lane 1). The TUAS activity is indicated with an arrow. (b) SDS-PAGE of *M. luteus *TUAS. Protein bands separated by 9.5% SDS-PAGE were visualized by silver staining. Lane 1: Molecular-mass standards of 97.4, 66.2, 42.7, and 31 kDa, respectively (top to bottom). Lane 2: Detergent extraction of membrane fraction. Lane 3: Protein after DEAE-cellulose column chromatography. Lane 4: TUAS purified to near homogeneity by isoelectric precipitation (pH 5.0) after DEAE-cellulose, gel filtration and adsorbent column chromatography. TUAS has two subunits A and B, upper subunit (A) is equivalent to 54 kDa and the lower subunit (B) to 52.5 kDa.

The TUAS activity is always associated with a large protein of molecular mass ~440 KDa (Figure 3(a)). This protein can be further separated by SDS PAGE into two small protein subunits A and B with molecular mass equivalent to 54 kDa and 52.5 KDa (Figure 3(b)). No TUAS activity was detected from these two small proteins even after SDS removal. Enzymatic activity was detected from a protein band on a native gradient PAGE. The band did not look very sharp. When exposed to prolonged electrophoresis, the TUAS band became fainter and fainter, while a wavy band appeared and migrated to the bottom of the gel. The wavy band did not have enzymatic activity but contained the 54 kDa and 52.5 kDa subunits. This result indicated that TUAS is an enzyme complex composed of an octomer (two tetramers), which assembled in a highly ordered fashion. When the 8 subunits disassociated, the protein lost its conformation as well as the catalytic function for TUA biosynthesis.

### 3.3. TUAS Possesses Hydrophobic Properties and Its Function Is Associated with the Cytoplasmic Membrane

**(a) Low Density**

Fractionation of Thesit-solubilized membrane fraction with ammonium sulfate (25%, w/v) results in the formation of a protein precipitate which can be collected by sedimentation. But very little TUAS activity (5%) was detected from the pellet or from the soluble supernatant (16%). The majority (70%) of the enzymatic activity was found in a low density layer which rises to the top of the tube during centrifugation. This layer contained detergent and lipids derived from the membrane as well as TUAS. The specific activity of TUAS in the lipid/detergent layer was increased by more than 3-fold over the original detergent-solubilized membranes (Table 1).

**(b) Pigments and Lipids**

The active fractions of TUAS are usually associated with a yellow color. Carotenoids are the characteristic pigments of the cytoplasmic membrane of *M. luteus* [12]. Even after extensive purification, the TUAS contains a pigment which is visually observed as a yellow-green color with absorption maxima at 300, 385, and 415 nm. The pigments and membrane lipids [13] are readily extracted from the enzyme by organic solvents such as chloroform and 1-butanol. However the enzyme lost activity after solvent extraction.

**(c) Formation of Vesicles or Micelles**

The TUAS possesses unusual hydrophobic properties which suggest that it readily forms vesicles. Fractionation of detergent-solubilized samples by gel filtration has been uniformly disappointing. Usually the amount of enzyme activity recovered is severely reduced. When activity is recovered, the elution position has always been at the excluded volume of the column, suggesting that the TUAS is not behaving as a soluble protein but rather exists in micelles or vesicles. Pigmented vesicles or micelles were observed through all purification and characterization procedures for this TUAS study.

**(d) Effect of Phospholipase C**

**Figure 4:**
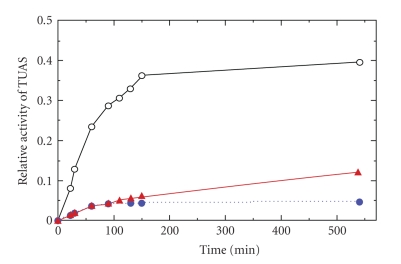
Time course of TUAS activity of *M. luteus* and the effect of phospholipase C on the activity. Open circle: TUAS activity; Closed circle: TUAS treated with phospholipase C; Triangle: addition of artificial phospholipid vesicles to TUAS reaction mix following phospholipase treatment.

To understand whether the membrane lipids associated with the enzyme are essential for TUAS activity, the purified TUAS was treated with exogenous phospholipase C. The time course in Figure 4 showed a rapid (15 minutes) and significant (~90%) inhibition of TUAS activity by phospholipase C. After 60 minutes of phospholipase treatment, artificial phospholipid vesicles (Sigma) were added to the TUAS reaction mix. Interestingly, a partial recovery of TUAS activity was observed (Figure 4). These results suggest that the membrane lipids associated or surrounding the TUAS enzyme complex play an important role in enzyme catalytic function.

**(e) TUAS Activity at Different Subcellular Compartments and Detergent Enhancement**

**Figure 5:**
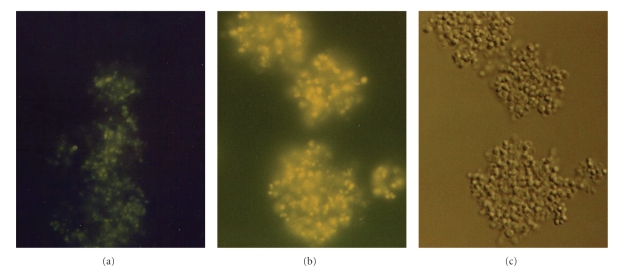
Immunoflluorescent images of *M. Luteus*. For (a) and (b), cells were observed under a fluorescent microscope with excitation wavelength 495 nm and emission wavelength 520 nm. (a) a negative control in which *M. luteus* cells were incubated with the secondary antibody conjugated to FITC. (b) *M. luteus* cells were incubated with both anti-TUAS serum and the secondary antibody conjugated to FITC. (c) The same bacterial cells as (b) except under visible light.

Four subcellular compartments of *M. luteus* were prepared by physical grinding (without using lysozyme). These included cytoplasm, cytoplasmic membrane, cell wall, and culture supernatant (cell filtrate). The fractions were standardized based on the protein concentration. The enzymatic activity of TUAS was analyzed in those fractions and control medium (before culturing bacteria).  Table 2 is the distribution of specific activity, in which cytoplasmic membrane contained the highest total and specific TUAS activity. The enzyme activity was also found in the cell wall fraction (6 units/mg of protein). Very little activity could be detected in cytoplasmic fractions. The culture supernatant contained yellow colored membrane vesicles and displayed a higher specific activity (8.7 units/mg of protein) of TUAS indicating that even in intercellular space, TUAS is embedded in the vesicle membrane and capable of catalyzing the polymerization of TUA polymer. However, it is unclear whether this is a life cycle dependent process. The TUAS is readily solubilized from membrane fragments by washing with 3 to 20% detergent, such as Thesit or CHAPS. Application of detergent also increases the specific activity in some of the cellular fractions. Antiserum to TUAS was raised in rabbits. Immunoblotting showed that the enzyme was mainly associated with the membrane fraction.  Figure 5 showed that intact *M. luteus* cells bound substantial amounts of the antibody as detected with an immunofluorescent assay, indicating that some TUAS is readily detectable from outside the cell wall as an extracellular protein.

## 4. Discussion

Bacterial surface polysaccharides are essential for bacterial survival. They play important roles including protection of the bacterial cell, signal transduction, nutrition regulation, and interaction with the environment as well as the host defense system. Consequently, there is increased interest in understanding the unique polysaccharide structures responsible for such interactions, as well as their biosynthetic origin, and ultimately, the mechanisms which control and regulate their synthesis. Recently much effort has been directed toward identification of the genes and gene products responsible for cell surface polysaccharide biosynthesis [14–16].

*M. luteus* is not a severe pathogenic bacterium, however for many years it has served as the model system for bacterial cell wall study. The TUA polymer of *M. luteus* is acidic and has some similarity with that of the so-called capsular polysaccharides (CPS) which are usually considered to be essential and virulent factors for pathogenic bacteria, such as Streptococca and Staphylococca [16–19].

**Figure 6:**
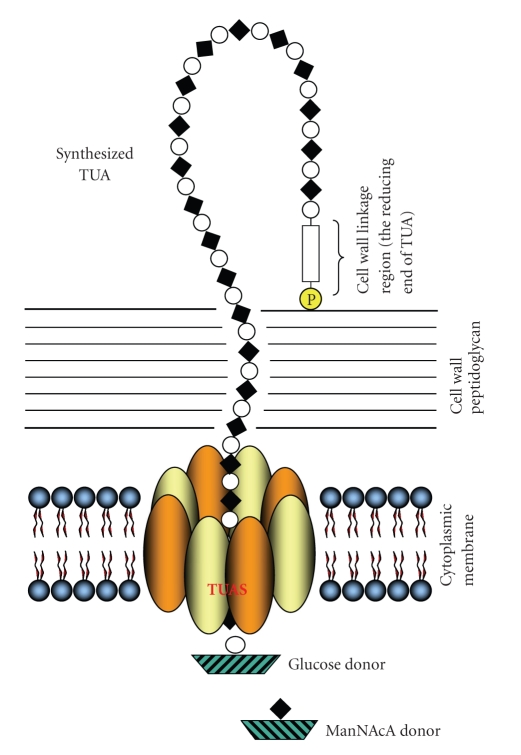
Proposed mode of action and subcellular location for cell wall TUA biosynthesis, in which an enzyme complex, TUAS, consisting of two kinds of enzymes (Glucosyltransferase and ManNAcA-transferase), serves as a multitasking polysaccharide assembling station on the bacterial membrane for efficient elongation of cell wall teichuronic acid. Black Diamond: ManNAcA, White Circle: glucose, Rectangle: linkage oligosaccharide, and Yellow Circle: phosphate.

Biosynthesis of polysaccharide requires glycosyltransferases, each of which transfers a specific sugar from an activated donor molecule, usually a nucleoside-diphospho-sugar, such as UDP-glucose, to an acceptor. Synthesis occurs either by sequential addition of residues to a growing polysaccharide chain or by assembly on a carrier lipid [20, 21] of an oligosaccharide unit which subsequently is polymerized. These enzymes are likely located in the cytoplasmic membrane. Although the activities of many such enzymes have been known for decades, and many genes of glycosyltransferases have been identified and expressed, purification of such functional membrane-associated enzymes in cell-free systems has progressed slowly. While many bacterial glycosyltransferase genes were identified through phenotype assays or homology searches, their gene products were, unfortunately, very hard to express as active enzymes. This may be due to a lack of the secondary modifications of the gene products, and the complexity of polysaccharide biosynthesis. In this study, through a delicate enzymologic approach, a membrane associated enzyme, teichuronic acid synthetase (TUAS), was purified from a gram-positive bacteria, *Micrococcus luteus. *Based on the experimental data obtained through this study, a mode of action model is proposed in Figure 6, which gives a unique interpretation of the topology of bacterial surface polysaccharide biosynthesis. 

The features of this model are as follows. (1) The four subunits of the Glucosyltransferase and the four subunits of the ManNAcA-transferase are assembled (in an alternating order) into an octomer, which forms a circular enzyme complex arranged like the segments of an orange. (2) The octomeric enzyme complex resides in the cytoplasmic membrane of gram-positive bacteria, with the catalytic domain in the center. (3) The enzyme can accept the sugar donors of either NDP-sugars or glycosyl carrier lipids [20, 21] from the cytoplasmic side and link the sugar to the nonreducing terminus of the existing TUA polymer, which is presumably covalently anchored to the cell wall peptidoglycan through an oligosaccharide and a phosphodiester bond. The reducing terminus of the linkage region, or that of the entire TUA chain, is a GlcNAc residue [3, 4]. (4) Each subunit of the enzyme is likely capable of adding one corresponding sugar, so after one round of catalysis, 8 sugars or an octosaccharide will be added to the existing polysaccharide chain and be squeezed out of the cytoplasmic membrane to the cell wall region or extracellular space. (5) In this model, no transporter, membrane porins and pumps are required. The TUAS is able to transfer two different sugars in alternating order, polymerize the disaccharide repeating units, transport the synthesized chain to the cell wall and protect the membrane integrity. The TUAS enzyme complex together with the surrounding membrane lipids serves as an efficient multitasking polysaccharide assembling station on the bacterial membrane for efficient elongation of cell wall teichuronic acid (Figure 6). 

Further study is needed to understand the assembling of the enzyme complex, the TUA polymer's attachment to the cell wall, and the other important component factors within the complex, as well as the expression and regulation of the gene operon encoded for TUAS. This knowledge will be very useful and important in understanding the mechanism of extracellular polysaccharide biosynthesis, as well as developing antibiotics against similar enzymes in pathogenic gram-positive bacteria other than *M. luteus.*

## Figures and Tables

**Table 1:** TUAS activity found in fractions after partitioning with 25% ammonium sulfate.

			TUAS		
Fraction	Activity		protein		Specific activity
	(units)	(%)	(mg)	(%)	(units/mg protein)
Original extract	126	100	18.0	100	7.0
Top lipid layer	89	71	3.8	21	23.0
Supernatant fraction	20	16	9.6	53	2.1
Precipitate (pellet)	6	5	4.7	26	1.3

**Table 2:** Distribution of TUAS activity in subcellular extract of *M. luteus. *

Cellular compartment	Specific activity (units*/mg protein)
Control medium	0
Culture supernatant	8.7
Cytoplasm (S-20 fraction)	0.5
Cell wall**	6.2
Cytoplasmic membrane**	19.9

* 1 Unit = 1 nmole [^14^C]glucose incorporated in 60 minutes in standard assay.

** Separated by sucrose density gradient centrifugation.
